# Regulation of Toll-like Receptor Signaling by the SF3a mRNA Splicing Complex

**DOI:** 10.1371/journal.pgen.1004932

**Published:** 2015-02-06

**Authors:** Brian P. O’Connor, Thomas Danhorn, Lesly De Arras, Brenna R. Flatley, Roland A. Marcus, Eveline Farias-Hesson, Sonia M. Leach, Scott Alper

**Affiliations:** 1 Department of Pediatrics, National Jewish Health, Denver, Colorado, United States of America; 2 Integrated Center for Genes, Environment and Health, National Jewish Health, Denver, Colorado, United States of America; 3 Department of Biomedical Research, National Jewish Health, Denver, Colorado, United States of America; 4 Department of Immunology and Microbiology, University of Colorado, Aurora, Colorado, United States of America; MD Anderson Cancer Center, UNITED STATES

## Abstract

The innate immune response plays a key role in fighting infection by activating inflammation and stimulating the adaptive immune response. However, chronic activation of innate immunity can contribute to the pathogenesis of many diseases with an inflammatory component. Thus, various negatively acting factors turn off innate immunity subsequent to its activation to ensure that inflammation is self-limiting and to prevent inflammatory disease. These negatively acting pathways include the production of inhibitory acting alternate proteins encoded by alternative mRNA splice forms of genes in Toll-like receptor (TLR) signaling pathways. We previously found that the SF3a mRNA splicing complex was required for a robust innate immune response; SF3a acts to promote inflammation in part by inhibiting the production of a negatively acting splice form of the TLR signaling adaptor MyD88. Here we inhibit SF3a1 using RNAi and subsequently perform an RNAseq study to identify the full complement of genes and splicing events regulated by SF3a in murine macrophages. Surprisingly, in macrophages, SF3a has significant preference for mRNA splicing events within innate immune signaling pathways compared with other biological pathways, thereby affecting the splicing of specific genes in the TLR signaling pathway to modulate the innate immune response.

## Author Summary

Within minutes after we are exposed to pathogens, our bodies react with a rapid response known as the “innate immune response.” This arm of the immune response regulates the process of inflammation, in which various immune cells are recruited to sites of infection and are activated to produce a host of antimicrobial compounds. This response is critical to fight infection. However, this response, if it is activated too strongly or if it becomes chronic, can do damage and can contribute to numerous very common diseases ranging from atherosclerosis to asthma to cancer. Thus it is essential that this response be tightly regulated, turned on when we have an infection, and turned off when not needed. We are investigating a mechanism that helps turn off this response, to ensure that inflammation is limited to prevent inflammatory disease. This mechanism involves the production of alternate forms of RNAs and proteins that control inflammation. We have discovered that a protein known as SF3a1 can regulate the expression of these alternate inhibitory RNA forms and are investigating how to use this knowledge to better control inflammation.

## Introduction

While the innate immune response plays a critical role in fighting infection, overactive or chronically activated innate immunity can contribute to many diseases with an inflammatory component [1–4]. Thus to fight infection without inducing inflammatory disease, a complex regulatory system has evolved to activate innate immunity when humans are exposed to pathogens and then turn the system off after a period of time to ensure that it is self-limiting. One family of innate immune receptors that senses pathogenic components is the Toll-like receptor (TLR) family. Different TLRs respond to different pathogenic stimuli; for example, TLR4 is activated in the presence of lipopolysaccharide (LPS) from Gram negative bacteria [5,6]. Binding of LPS to TLR4 and its co-receptor MD-2 leads to recruitment and activation of the signaling adaptor MyD88, which in turn recruits a family of related kinases: IRAK4, IRAK1, and IRAK2 [7]. This signaling cascade continues, culminating in the activation of the transcription factor NFκB and the activation of several MAP kinase pathways [7]. This in turn leads to the production of, among other things, inflammatory cytokines.

One mechanism that has evolved to ensure that TLR4 activation is self-limiting is the feedback-induced production of a variety of negative regulators of TLR signaling [8–14] including the production of alternatively spliced forms of TLR signaling components [15–25]. For example, while the LPS receptor TLR4 is encoded by a three exon mRNA, an alternately spliced mRNA that includes an extra exon between exons two and three has been identified [18]. This extra exon introduces a premature stop codon, resulting in the production of a soluble fragment of TLR4 (sTLR4) that can bind LPS but that cannot signal to the downstream components of the pathway. Thus, sTLR4 acts as a dominant inhibitor of TLR signaling [18]. Similarly, negatively acting splice forms of MD-2, MyD88, IRAK1, IRAK2, and many other TLR signaling components have been described [15–25]. The production of many of these negatively acting alternate splice forms is induced by LPS stimulation [16–19], suggesting that the inflammatory stimulus mediates its own negative feedback loop to limit the innate immune response, thereby preventing inflammatory disease.

While RNAseq and individual gene studies have determined that alternative splicing is an important regulatory mechanism to control TLR signaling, thus far there has been only limited investigation of how this alternative pre-mRNA splicing is regulated. We have identified the SF3a and SF3b mRNA splicing complexes as novel regulators of innate immunity [26,27]. These mRNA splicing complexes bind to the U2 small nuclear ribonucleoprotein (snRNP), which in turn binds to the branch site near the 3’ end of introns to control mRNA splicing with the rest of the spliceosome [28–34]. Weakening of U2 snRNP activity is expected to perturb mRNA splicing, causing exon skipping or intron retention [35–38]. We found that inhibition of SF3a or SF3b by RNAi or a pharmacological agent in mouse or human macrophages weakened the innate immune response induced by several TLR agonists including LPS [26,27]. In particular, SF3a1 inhibition diminished the LPS-induced production of IL-6, TNFα, RANTES, and IL-10 [27]. Importantly, this effect on innate immunity occurred at a level of gene inhibition (roughly 80%) that did not affect general cell functions such as viability or phagocytosis [26]. This suggests that inflammatory signaling pathways may be more sensitive to perturbation of the spliceosome than other pathways. Consistent with this theory, RNAi-mediated inhibition of Eftud2, which functions with the U5 snRNP at a later stage of spliceosome assembly [30,39–45], also weakened the innate immune response to LPS without affecting cell viability [46]; in contrast, overexpression of Eftud2 increased the response to LPS [46].

The effects of these splicing factors on innate immunity are mediated in part by control of alternative splicing of MyD88 [26,46]. MyD88 is encoded by a five-exon mRNA (long form or MyD88_L_) that encodes the positively acting TLR signaling adaptor. A shorter mRNA lacking exon 2 (MyD88_S_) encodes a dominantly acting negative regulator of TLR signaling that prevents IRAK activation [15,19,20]. Inhibition of SF3a, SF3b, or Eftud2 leads to an increase in the production of MyD88_S_, which in part explains the effect of these mRNA splicing genes on innate immunity [26,46]. However, our data indicated that other TLR signaling components also likely mediate the effects of mRNA splicing genes on innate immunity [26]. Based on these data, we have hypothesized that the splice site choices in MyD88 and perhaps other TLR signaling genes have evolved to be exquisitely sensitive to cellular conditions because of their functional significance, and may be key regulatory points of a mechanism to limit inflammation.

To better understand the effects of the spliceosome on TLR signaling, we now use RNAseq to examine the full complement of genes and splicing events regulated by the SF3a complex in mouse macrophages. We find that key cis-acting regulatory sequences mediate the effects of SF3a on alternative splicing. In keeping with our hypothesis, pathway analyses of these data indicate that TLR signaling and other innate immune signaling pathways are among the most sensitive pathways to inhibition of SF3a1 in macrophages. We find several genes in TLR pathways whose expression or mRNA splicing are altered by SF3a1 inhibition. These include the production of the known negative regulatory splice form of TLR4 as well as a newly identified negatively acting splice form of IKKβ. Thus, SF3a1 regulates innate immunity by controlling multiple mRNA splicing events in TLR signaling pathways in macrophages.

## Results

### Strategy to analyze the effects of SF3a1 inhibition

**Figure 1:**
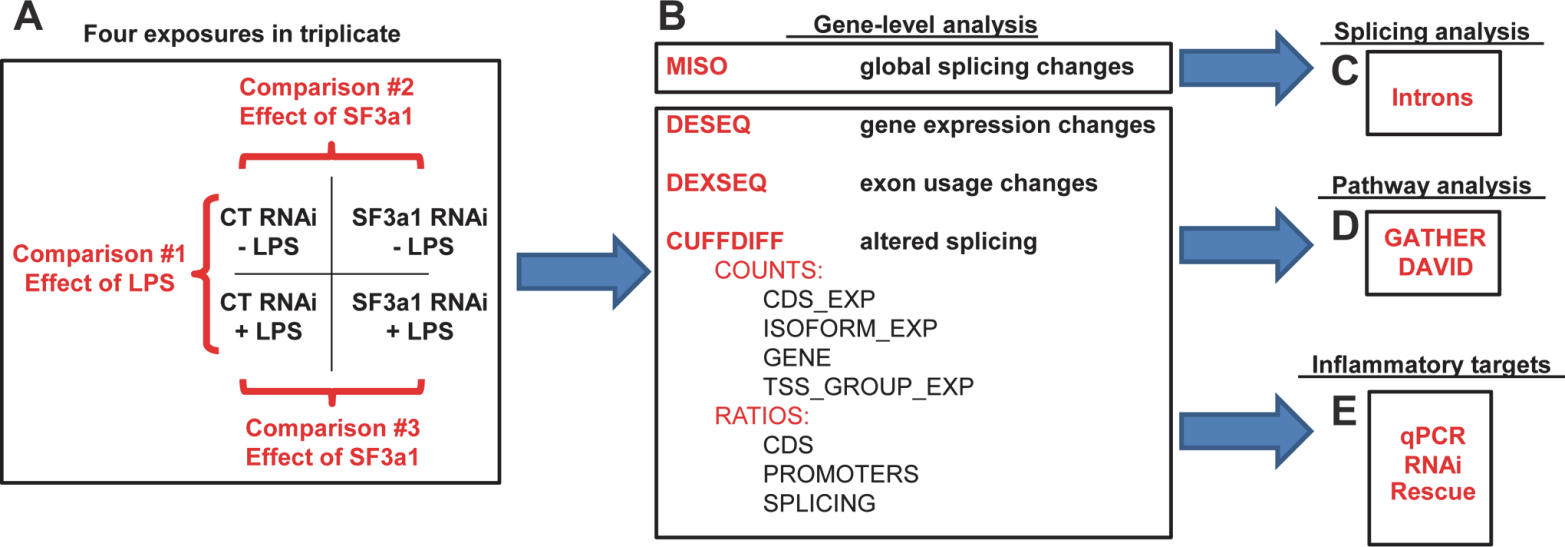
Plan to investigate the effect of SF3a1 inhibition. The schematic depicts our experimental approach for investigating the effect of SF3a1 inhibition on mRNA splicing and innate immunity. Mouse RAW264.7 cells were treated with either SF3a1 siRNA or control siRNA and subsequently exposed (or not) to LPS. These mRNA samples were then subjected to poly-A mRNA sequencing (A). The sequence data were then analyzed using MISO (B) to determine the global effects of SF3a1 on alternative pre-mRNA splicing and to investigate intron sequences that mediated these alternative splicing effects (C). The sequence data also were analyzed using DESeq, DEXSeq, and Cuffdiff to identify genes and isoforms whose expression was regulated by SF3a1 (B). This gene- and isoform-level analysis was in turn used for pathway analysis (D) and to identify specific TLR signaling pathway genes that mediate the effects of SF3a on innate immunity (E).

A schematic outlining our experimental strategy is depicted in Fig. 1. To test the effect of SF3a1 inhibition, the RAW264.7 mouse macrophage cell line was treated with either SF3a1 siRNA or control non-targeting siRNA. Following siRNA treatment, the cells were exposed for four hours to either 20 ng/ml LPS or no LPS as a control. All siRNA treatments and subsequent LPS exposures were performed in triplicate, resulting in 12 total samples analyzed by RNAseq (Fig. 1A). Following the LPS exposures, supernatant was collected for ELISA analysis to verify that, as expected, LPS induced IL-6 production and that SF3a1 siRNA treatment inhibited LPS-induced IL-6 production. RNA was purified from the adherent cells for qPCR analysis to verify SF3a1 gene knockdown (∼80%) and for RNAseq analysis. No effects on viability were observed at this level of knockdown [26].

Three different experimental comparisons were monitored (Fig. 1A): (1) the effect of LPS was monitored by comparing the effects of control siRNA treatment in either the absence or presence of LPS; (2) the effect of SF3a1 inhibition in the absence of LPS; and (3) the effect of SF3a1 inhibition in the presence of LPS. Several computational approaches were taken for this analysis as outlined below.

To investigate the global effects of SF3a on mRNA splicing, we used the MISO [47] software package (Fig. 1B, S1–S5 Tables). MISO identifies changes in mRNA splicing by mapping RNAseq data onto pre-identified intron and exon isoform structures from a subset of genes. These data were in turn used for computational analyses of intron and exon sequences that regulate mRNA splicing (Fig. 1C).

To determine how SF3a affects innate immunity, three different software packages (DESeq, DEXSeq, and Cufflinks) were used to identify genes and gene isoforms whose expression was regulated by SF3a (Fig. 1B). DESeq [48] maps RNAseq data onto pre-identified gene structures. Thus this gene-level analysis can be used to identify changes in total expression of each gene (S6–S8 Tables), but cannot identify changes in isoform usage. In contrast, DEXSeq [49], which performs an exon-by-exon level analysis of RNAseq data, was used to identify changes in exon expression and therefore isoform usage (S9–S11 Tables). Finally, Cufflinks [50,51], which unlike the other software packages that compare sequence data to known transcripts, analyzes the sequence data *de novo* to identify both known and novel transcripts, which can then be compared between experiments using Cuffdiff (S12–S18 Tables). These gene and isoform lists were then used to inform pathway analysis with the GATHER [52] and DAVID [53,54] software tools (Fig. 1D) and also were used to identify genes responsible for mediating the effects of splicing factors on innate immunity (Fig. 1E).

### Effects of LPS on gene expression and alternative splicing

As expected, treatment with LPS increased mRNA levels for numerous cytokines and chemokines (S6 Table) including but not limited to TNFα, IL-6, IL-1β, and IL-12. Among the top pathways altered by LPS at both the gene level (S6 Table) and exon level (S9 Table) were innate immune signaling pathways: TLR signaling, cytokine-cytokine receptor signaling, and MAP Kinase signaling (Table 1). Thus, LPS stimulation alters the expression of LPS-response genes at both the gene and isoform levels.

**Table 1:** Pathways affected by LPS Treatment.

**DESeq**			
**#**	**Annotation** *	**Genes in pathways**	**P-value**
1	path:mmu04060: Cytokine-cytokine receptor interaction	146	0.0010
2	path:mmu00190: Oxidative phosphorylation	5	0.0014
3	path:mmu04010: MAPK signaling pathway	124	0.0087
4	path:mmu04510: Focal adhesion	111	0.0166
5	path:mmu04620: Toll-like receptor signaling pathway	56	0.0215
**DEXSeq**			
1	path:mmu04210: Apoptosis	31	0.0002
2	path:mmu04010: MAPK signaling pathway	28	0.0094
3	path:mmu04060: Cytokine-cytokine receptor interaction	22	0.0324
4	path:mmu05050: Dentatorubropallidoluysian atrophy (DRPLA)	5	0.0362
5	path:mmu04620: Toll-like receptor signaling pathway	12	0.0384

*Innate immune signaling pathways underlined.

### Effects of SF3a1 on alternative splicing

**Figure 2:**
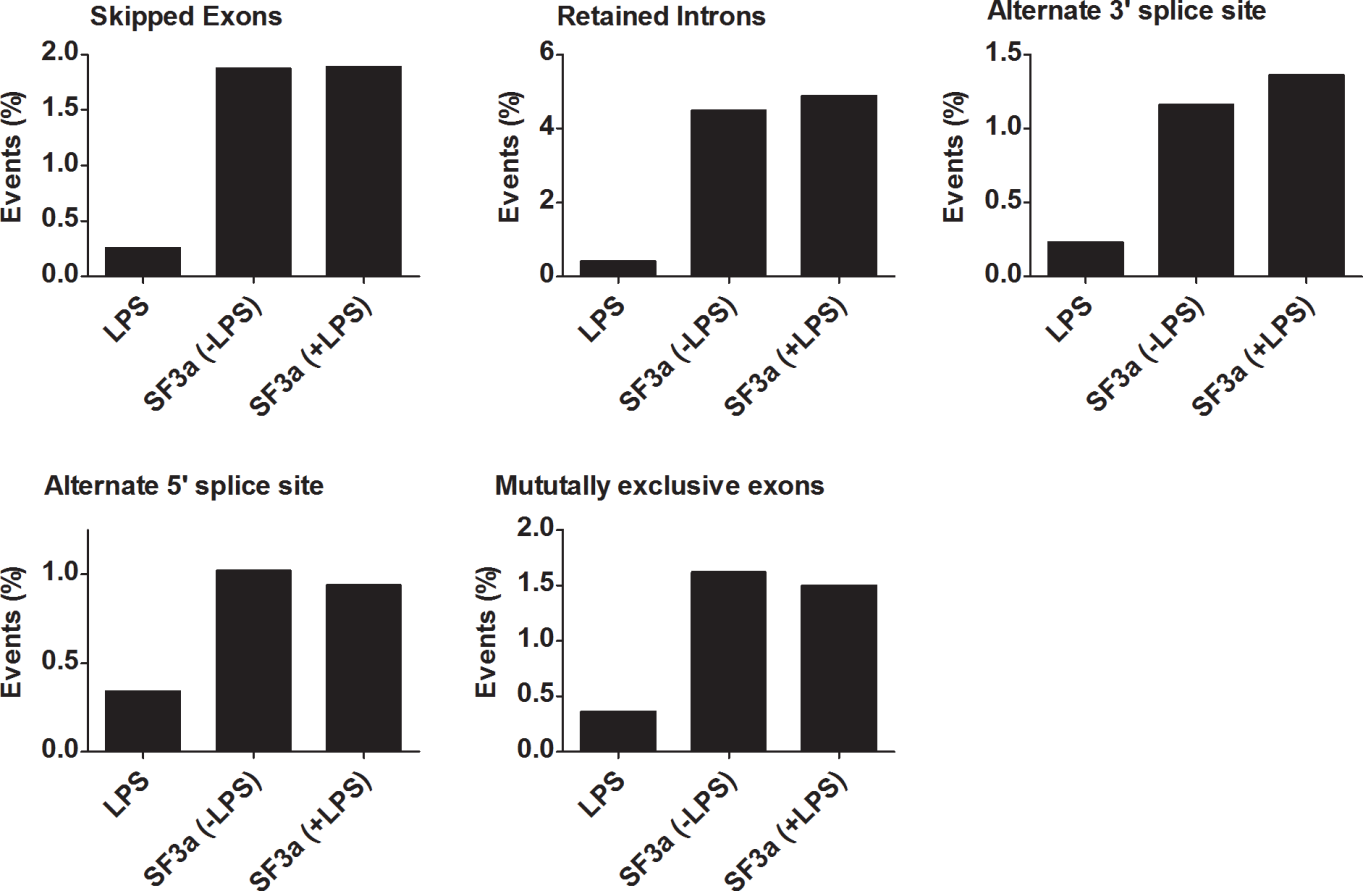
SF3a1 regulates multiple modes of alternative pre-mRNA splicing. The figure depicts the percentage of possible alternative splicing events (intron retention, exon skipping, alternate 3’ and 5’ splice site choice, and mutually exclusive exon usage) affected by either LPS treatment or SF3a1 inhibition (in the absence or presence of LPS) in the RAW264.7 cell line. Complete data depicted in S19 Table.

SF3a1 is an essential mRNA splicing factor, and as such, its inhibition is expected to alter mRNA splicing. Using MISO, we determined that SF3a1 inhibition, in either the absence or presence of LPS, affected multiple classes of alternative splicing events (Fig. 2, S19 Table), including intron retention, exon skipping, alternate 3’ and 5’ splice site usage, and altered mutually exclusive exon usage. In particular, a large number of intron retention and exon skipping events were identified by this analysis. In contrast, LPS stimulation affected all classes of splicing changes but did so at much lower frequency.

### Cis-acting sequences render certain alternative splicing events particularly sensitive to SF3a inhibition

While SF3a1 inhibition affected numerous alternative pre-mRNA splicing events (Fig. 2), the vast majority of potential mRNA splicing events in macrophages were not significantly affected even though SF3a1 levels are at only 20% of their wild type levels in these studies. What renders some splice site choices so sensitive to SF3a inhibition? To answer this question, we investigated intron sequences known to regulate mRNA splicing. Intron sequences that govern splicing include the GT at the 5’ splice site, the AG at the 3’ splice site, the polypyrimidine tract that is located just upstream of the 3’ splice site, and the branch site located still further upstream [55]. Assembly of splicing regulators at the 3’ splice site involves binding of the SF1 protein to the branch site [56–58] and the U2AF1/2 complex to the polypyrimidine tract and 3’ splice site [59–62]. This facilitates the recruitment of the U2 small nuclear ribonucleoprotein (snRNP), which binds to the branch site. Activation of the U2 snRNP additionally requires two accessory protein complexes, SF3a and SF3b [31–33,63–65].

**Figure 3:**
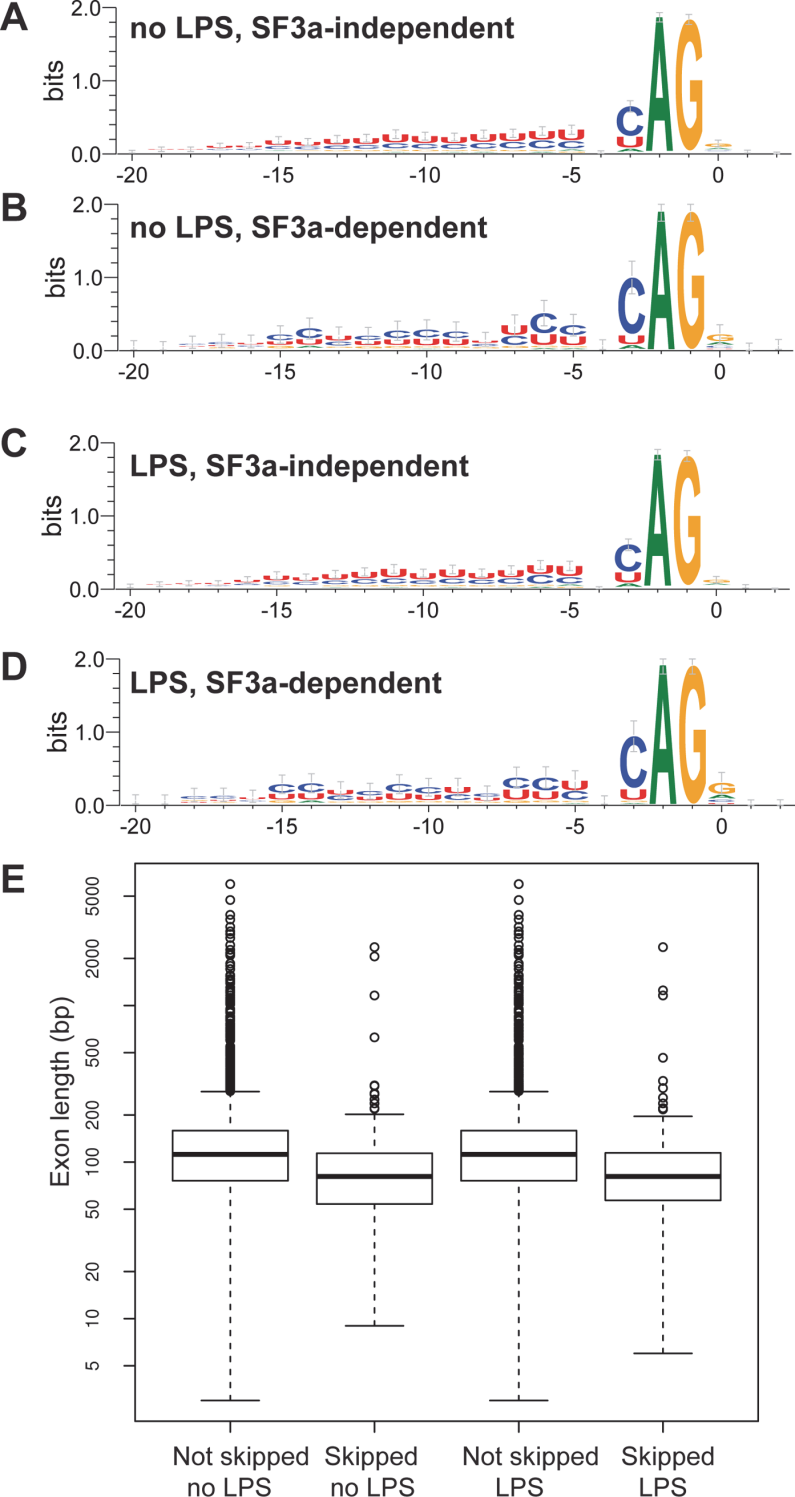
Identification of intron and exon features that correlate with alternative splicing events regulated by SF3a1. (A-D) Sequence logo plots of the polypyrimidine tracts and 3’ splice site of introns that are retained when SF3a1 is inhibited (SF3a-dependent) or introns that are spliced out correctly when SF3a1 is inhibited (SF3a-independent, or at least less dependent) in the RAW264.7 cell line. (E) The length of exons that are skipped when SF3a1 is inhibited compared to those exons that are not skipped. The boxplots were generated using default conventions in R 3.0.1. The boxes show the interquartile range, the whiskers the most extreme data point that is no more than 1.5 times the interquartile range from the box. Exon lengths are depicted on a log scale. “Skipped” refers to exons with increased rate of skipping when SF3a1 is inhibited, whereas “not skipped” refers to exons that have the same rate of skipping, regardless of SF3a1 levels.

We used MISO to identify introns that were retained when SF3a1 was inhibited (SF3a-“dependent” introns) and introns that were spliced out normally when SF3a1 was inhibited (SF3a-“independent” or at least “less dependent” introns) and subsequently compared their sequences. Similarly, we compared introns upstream of exons that were skipped when SF3a1 was inhibited to downstream introns and to introns flanking exons that were not skipped, despite being annotated as potential candidates. We did not observe any significant differences in the nucleotides immediately surrounding the 5’ or the 3’ splice site when SF3a1 inhibition induced intron retention or exons skipping. However, we did observe differences in the polypyrimidine tracts of introns that were retained following SF3a inhibition (Fig. 3A–D). These introns (undergoing SF3a-dependent splicing) had a less U-rich and more C-rich polypyrimidine tract compared to introns that were not retained (SF3a-independent splicing) (Fig. 3A–D, raw data in S20 Table). In contrast, the polypyrimidine tracts in introns upstream of skipped exons were not significantly different from those in introns downstream of skipped exons. Moreover, these polypyrimidine tracts that flanked skipped exons were not significantly different from those that flanked non-skipped exons.

We also examined the length of introns and exons at alternatively spliced sites when SF3a1 was inhibited and found that skipped exons were shorter than non-skipped exons (Fig. 3E, mean length 114 skipped vs 150 non-skipped, p = 4×10^−15^, Mann-Whitney U-test). Moreover, as noted previously [66–68], we observed that exons were more likely than expected by chance (>33%) to be of a length that is a multiple of three base pairs, and skipped exons tended to be even more enriched for such “in-frame” exons (no LPS: 44.5% not skipped vs 56.3% skipped, p = 0.0020; with LPS: 45.0% not skipped vs 55.3% skipped, p = .0086, both Pearson’s χ^2^-test). Thus, skipped exons in genes frequently do not alter the reading frame of their encoded proteins, making it more likely that they will not completely abolish protein function.

### SF3a1 inhibition perturbs innate immune signaling pathways

**Figure 4:**
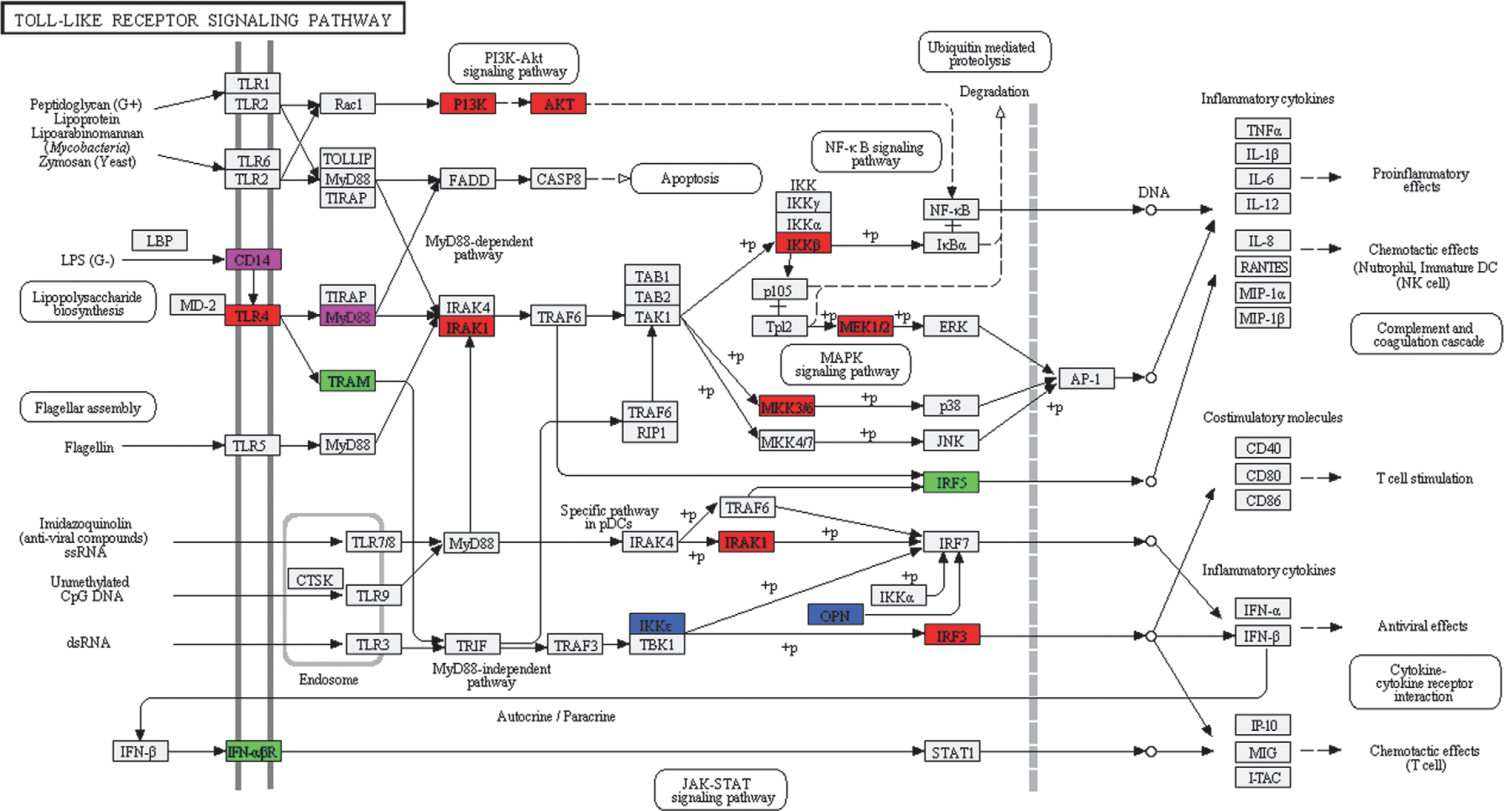
SF3a1 inhibition affects pre-mRNA splicing of many genes in the TLR signaling pathway. The schematic depicts the Toll-like receptor signaling pathway (KEGG map04620) [143,144] with genes whose splicing is altered (DEXSeq analysis) when SF3a1 is inhibited color coded as follows: red (altered in the absence or presence of LPS), green (only in the absence of LPS), blue (only in the presence of LPS). Additionally, altered splicing of two genes in purple was identified in our other analyses. Generated using DAVID [53,54].

As observed previously [26,27], inhibition of SF3a1 in the presence of LPS diminished production of numerous cytokines and chemokines (S8 Table), including but not limited to IL-6, CCL5 and IP10. We previously speculated that inflammatory processes in macrophages were more sensitive to perturbation of the spliceosome than are other pathways, because inhibition of splicing factors weakened innate immunity without significantly affecting macrophage viability or phagocytosis [26]. Consistent with this speculation, while many genes and pathways are affected by SF3a1 inhibition in macrophages, we find that innate immune signaling pathways are among the most significantly altered pathways at the level of mRNA splicing (DEXSeq analysis) when SF3a1 is inhibited, either in the absence or presence of LPS (Table 2). Examination of TLR signaling pathways identified several genes whose expression or splicing was altered by SF3a1 inhibition in the absence and/or presence of LPS (Fig. 4). We decided to investigate the effects of three of these genes in detail that function in the MyD88-NFκB arm of the LPS response pathway (Fig. 4). These three genes were the LPS receptor TLR4 and the downstream signaling kinases IRAK1 and IKKβ (alias IKBKB). TLR4, IRAK1, and IKKβ were identified by the DEXSeq analyses as alternatively spliced in both the absence and presence of LPS (S10–S11 Tables). IKKβ was additionally identified by one of the Cuffdiff analyses (S15 Table). All three of these genes are positive effectors of the innate immune response.

**Table 2:** Pathways affected by SF3a1 inhibition.

**No LPS**			
**#**	**Annotation** *	**Genes in pathway**	**P-value**
1	path:mmu04510: Focal adhesion	77	0.0039
2	path:mmu04210: Apoptosis	41	0.0062
3	path:mmu04010: MAPK signaling pathway	77	0.0069
4	path:mmu04910: Insulin signaling pathway	52	0.0077
5	path:mmu04620: Toll-like receptor signaling pathway	35	0.0228
6	path:mmu05010: Alzheimer’s disease	12	0.0460
**20 ng/ml LPS**			
1	path:mmu04010: MAPK signaling pathway	85	0.0036
2	path:mmu04210: Apoptosis	45	0.0036
3	path:mmu04510: Focal adhesion	78	0.0052
4	path:mmu04910: Insulin signaling pathway	50	0.0141
5	path:mmu04060: Cytokine-cytokine receptor interaction	73	0.0211
6	path:mmu04620: Toll-like receptor signaling pathway	35	0.0290

*Innate immune signaling pathways underlined.

Additionally, we chose to investigate two other genes that affect upstream components of the TLR4 signaling pathway that were not identified by DEXSeq but were identified in the other analyses. RAB7b controls the trafficking and subsequent destruction of TLR4 [69] and thus is a negative regulator of TLR signaling. CD14 functions to bring LPS to the TLR4 receptor and is a positive effector of TLR signaling [70]. Expression of RAB7b (alias 5430435G22Rik) was flagged as significantly increased in several analyses including DESeq (S7–S8 Tables) and Cuffdiff (S12 Table). CD14 was identified in Cuffdiff analyses that used mouse genome mm9 but was not identified as a significantly changed gene in these analyses using mouse genome mm10, possibly due to differences in CD14 gene annotation in the two databases.

### Intron retention in positively acting TLR4 pathway signaling genes when SF3a1 is inhibited

**Figure 5:**
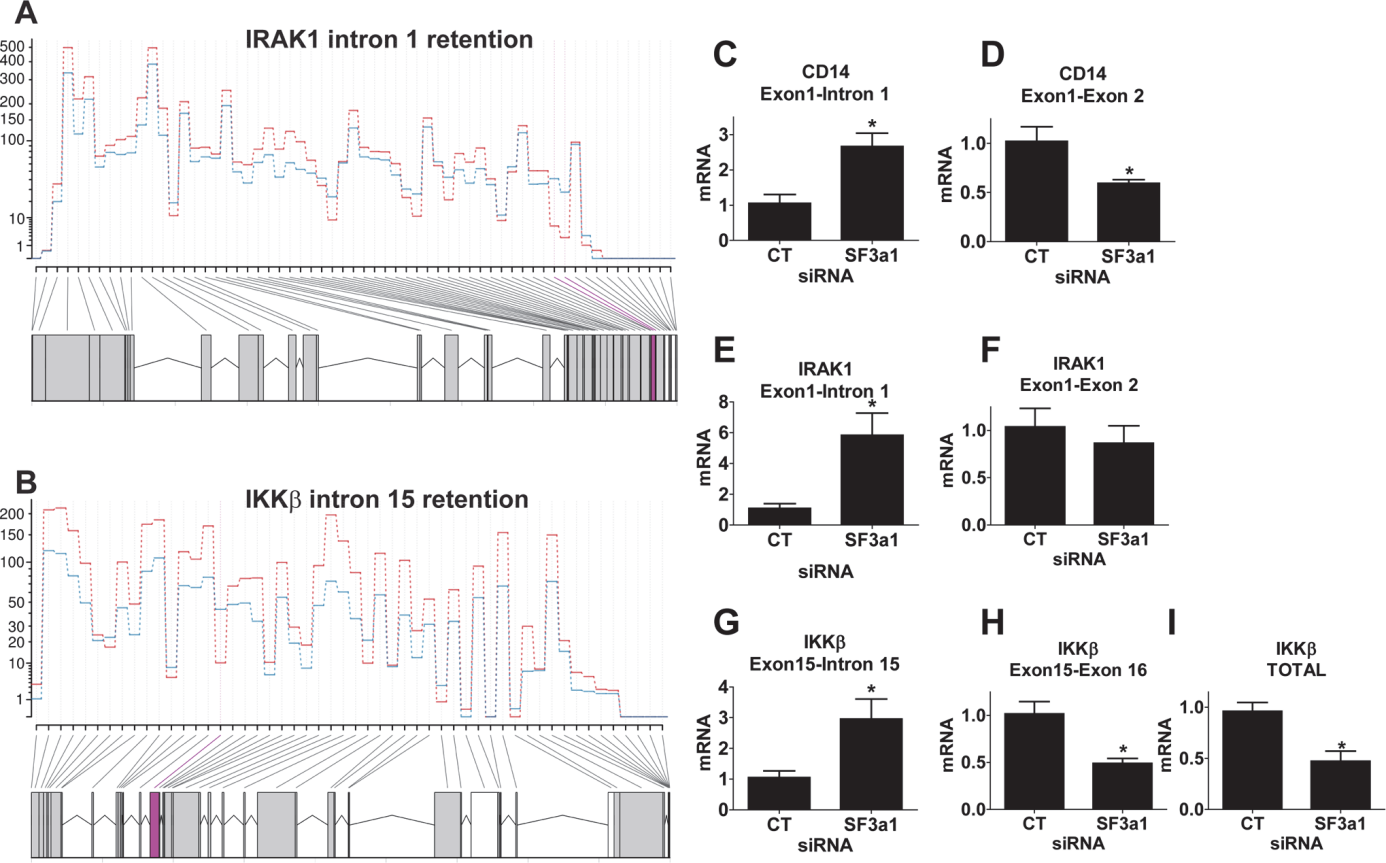
SF3a1 inhibition leads to intron retention in several TLR signaling pathway genes. (A,B) These panels depict sequence reads (average of 3 replicates, each; generated by DEXSeq) across IRAK1 or IKKβ (5’ end of gene on right, control siRNA in red, SF3a1 siRNA in blue). The retained introns (intron 1 in IRAK1 or intron 15 in IKKβ) are shaded in purple. (C-I) These panels display the results of qPCR assays on RAW264.7 cells used to monitor production of the indicated mRNA isoforms (expression normalized so that 1 is the expression in the presence of control siRNA). CT indicates control siRNA in this an all other figures. LPS exposures were performed for six hours in the presence of 20 ng/ml LPS. Asterisks indicate results that were statistically different than control in this and all other figures.

The RNAseq analysis indicated that three of these five genes had intron retention events when SF3a was inhibited: IRAK1 intron 1 (Fig. 5A), IKKβ intron 15 (Fig. 5B), and CD14 intron 1. While DEXSeq identifies alterations in exon expression in RNAseq data, in all these cases, DEXSeq also identified intron retention events due to reported non-canonical isoforms in Ensembl. To validate these RNAseq data, we monitored expression of the various gene isoforms using qPCR with isoform-specific primers. Moreover, we performed these qPCR studies on a second set of RNA samples from independent SF3a1 RNAi treatments and LPS exposures. In all three cases, we found that inhibition of SF3a1 in the presence of LPS led to increased retention of the expected intron and a concomitant decrease in the expression of the isoform that crossed that particular exon-exon junction (Fig. 5C–H). We also confirmed that the canonical IKKβ isoform was decreased following SF3a inhibition by using a second set of qPCR primers that lie further downstream in the gene (Fig. 5I). Thus, intron retention in these three genes diminishes production of the wild type, positively acting isoform. This is consistent with the effects of SF3a inhibition, which weakens innate immunity [26,27].

To confirm that these mRNA splicing changes were reflected at the protein level, we monitored the level of IRAK1 and IKKβ by western blot following SF3a1 siRNA treatment. Retention of intron 1 in IRAK1 is predicted to truncate the 750 amino acid protein after only 47 amino acids. Retention of intron 15 in IKKβ is predicted to truncate the 757 amino acid full length protein and generate a 555 amino acid protein containing the first 526 amino acids of IKKβ and 29 novel intron-encoded amino acids. Using antisera that recognize IRAK1 and IKKβ, we observed decreased levels of IRAK1 and IKKβ when SF3a1 was inhibited by RNAi (S1 Fig.) [note that IRAK1 levels were monitored in the absence of LPS as LPS exposure alters electrophoretic mobility and stability of IRAK1 [15,71–74]]. In contrast, SF3a1 inhibition did not affect production of βactin (S1 Fig.). We were not able to detect the predicted 555 amino acid truncated IKKβ, even on much longer exposures of the western blot. This may be because the relative levels of the proteins differ (which we cannot determine from the current qPCR data) or because the truncated protein is unstable.

**Figure 6:**
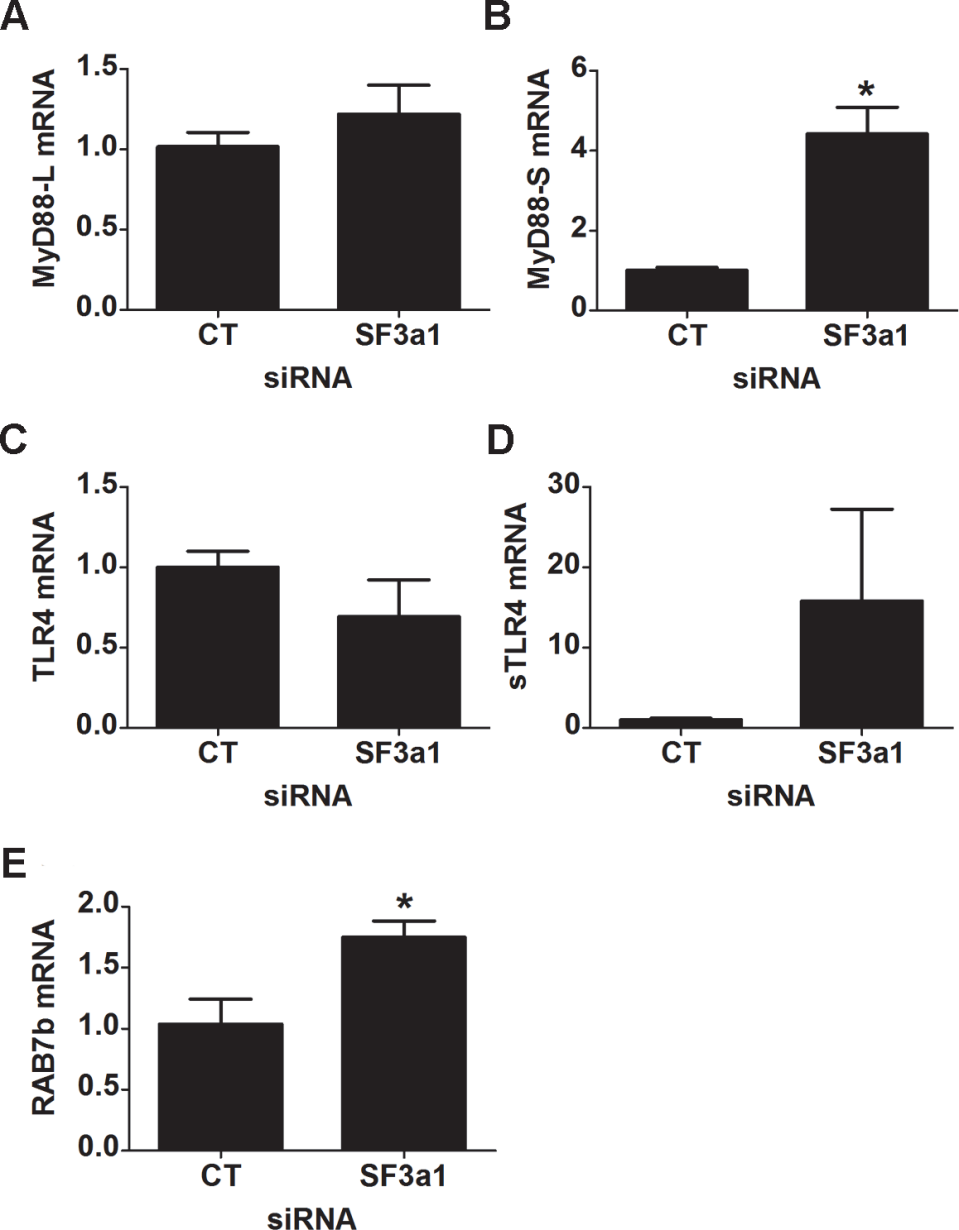
SF3a1 inhibition induces production of negatively acting splice forms of TLR signaling pathway genes. Panels A–E display the results of qPCR assays on RAW264.7 cells used to monitor production of the indicated mRNA isoforms (expression normalized so that 1 is the expression in the presence of control siRNA). CT indicates control siRNA in this an all other figures. LPS exposures were performed for six hours in the presence of 20 ng/ml LPS.

To test how general these effects were, we also monitored these intron retention events when SF3a1 was inhibited in a second mouse macrophage cell line, J774A.1. Inhibition of SF3a1 in J774A.1 cells also diminishes the innate immune response to LPS [27]. As observed previously [26], qPCR analysis indicated that expression of the negatively acting MyD88_S_ isoform was increased when SF3a1 was inhibited in RAW264.7 cells (Fig. 6A,B), and we find that MyD88_S_ is likewise increased following SF3a1 inhibition in J774A.1 cells (S2A–S2B Fig.). We found that some but not all of the effects of SF3a1 knockdown on intron retention events were recapitulated in J774A.1 cells. CD14 intron 1 was retained in J774A.1 cells following SF3a1 inhibition (S2C–S2D Fig.). We also observed a decrease in IRAK1 levels in J774A.1 cells following SF3a1 inhibition (S2E Fig.) but did not observe a concomitant increase in IRAK1 intron 1 retention (S2F Fig.). We did not detect intron 15 retention in IKKβ in J774A.1 cells when SF3a1 was inhibited with siRNA (S2G–S2H Fig.). Thus, some but not all of the altered splicing events detected in RAW264.7 cells were recapitulated in a second macrophage cell line J774A.1. The differences could reflect a difference in SF3a1 knockdown in the two cell lines.

### Increased expression of negatively acting factors when SF3a1 is inhibited

Despite our ability to detect alterations in MyD88_S_ by qPCR when SF3a1 is inhibited (Fig. 6A,B), we did not identify differential expression of MyD88_S_ in the current RNAseq study, likely because of the very small quantity of MyD88_S_ mRNA present in cells. The vast majority of sequence reads in MyD88 lie entirely within exons. These reads cannot distinguish between the two splice forms because they will be common to both MyD88_L_ and MyD88_S_; thus, only reads that cross the unique splice junctions in MyD88_L_ and MyD88_S_ will be informative as to the ratio of the two isoforms. Based on RT-PCR, we previously estimated that the ratio of MyD88_L_:MyD88_S_ was approximately 20:1 in unstimulated cells [26]. The current RNAseq data suggest that this ratio could even be larger; in unstimulated cells, we identified 282 reads that crossed the exon 1-exon 2 junction and 217 reads that crossed the exon 2-exon 3 junction (both of which are reads corresponding to MyD88_L_). In contrast, in unstimulated cells, we only obtained 7 reads that crossed the unique MyD88_S_ exon 1-exon 3 junction.

The RNAseq data also indicated that an alternative splice form of TLR4 was generated when SF3a1 was inhibited; this involved splicing of TLR4 to either of two alternative exons >70 kb downstream of TLR4. However, neither of these alternative splice forms has been identified in the plethora of previous studies on TLR4, and we were unable to obtain products corresponding to these computational predictions using RT-PCR. However, we did note that RNAseq reads were identified between exons 3 and 4 in TLR4 when SF3a1 is inhibited. An alternative splice form of TLR4 has been described previously in which an extra exon is incorporated between exons 3 and 4; this extra exon introduces a stop codon that produces a truncated soluble version of TLR4 (sTLR4) that acts as a negative regulator of signaling [18]. Using qPCR, we were able to verify that TLR4 levels were moderately decreased and sTLR4 levels were substantially increased when SF3a1 was inhibited (Fig. 6C,D).

Our RNAseq analysis also indicated that expression of the negative regulator RAB7b was increased when SF3a1 was inhibited, and we were able to verify this by qPCR (Fig. 6E). Thus, SF3a1 inhibition leads to increased expression of Rab7b and sTLR4, both negative regulators of TLR signaling.

**Figure 7:**
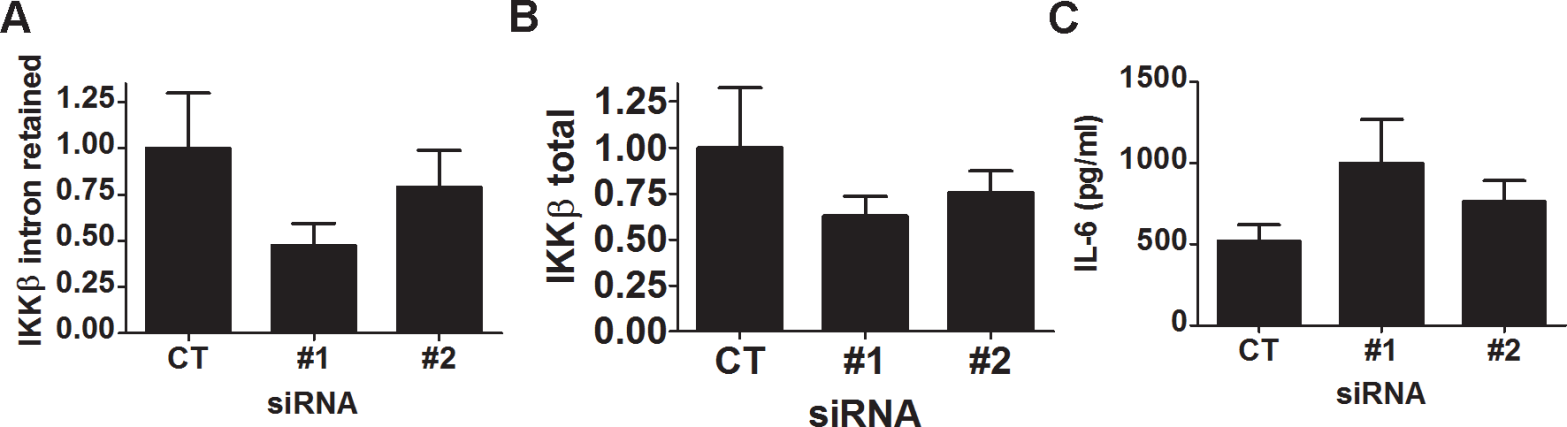
siRNAs targeting IKKβ intron 15 lead to increased production of LPS-induced IL-6. RAW264.7 macrophages were treated with either of 2 siRNAs targeting IKKβ intron 15 or control non-targeting siRNA, were subsequently exposed to 20 ng/ml for 6 hours, and then expression of both IKKβ isoforms was monitored by qPCR (A,B) and IL-6 production was monitored by ELISA (C).

As described above, inhibition of SF3a1 led to a decrease in production of the wild type IKKβ mRNA and an increase in an alternative mRNA form of IKKβ retaining intron 15 (Fig. 5B, G–I). cDNAs with similar intron 15 retention events also have been reported in humans (Ensembl transcript ENST00000520201, UCSC transcript uc010lxh.2, mRNA AB209090). While this alternate transcript also includes intron 14 (163 nt) in human, we see no evidence of intron 14 retention in our experiments with mouse. Retention of intron 15 in mouse results in a premature stop codon that truncates IKKβ after amino acid R526 plus 29 intron-encoded amino acids; this deletes the last 231 amino acids of IKKβ. The resulting protein contains the NH_2_-terminal kinase domain but lacks the COOH-terminal NEMO binding domain. IKKβ, IKKα, and NEMO together form a complex that phosphorylates IκBα and is thus critical for LPS-induced NFκB activation [75,76]. Interestingly, an alternative splice form of the related protein IKKε that is truncated in a similar location encodes a dominant negative signaling molecule that inhibits viral infection-induced activation of IRF3 and NFκB [77]. We therefore investigated if this truncated IKKβ (which we refer to as IKKβb) could likewise act in dominant negative fashion. We inhibited production of this alternatively spliced IKKβb mRNA using either of two different siRNA duplexes that target intron 15 in IKKβ. Both siRNAs decreased production of both IKKβ and IKKβb isoforms, with stronger inhibition of the IKKβb isoform (Fig. 7A,B), and increased LPS-induced IL-6 production (Fig. 7C). Inhibition of wild-type IKKβ should diminish LPS-induced cytokine production, so this increased IL-6 production is consistent with IKKβb being a novel inhibitory isoform.

### The effect of SF3a1 on innate immunity is mediated by several TLR signaling pathway genes

**Figure 8:**
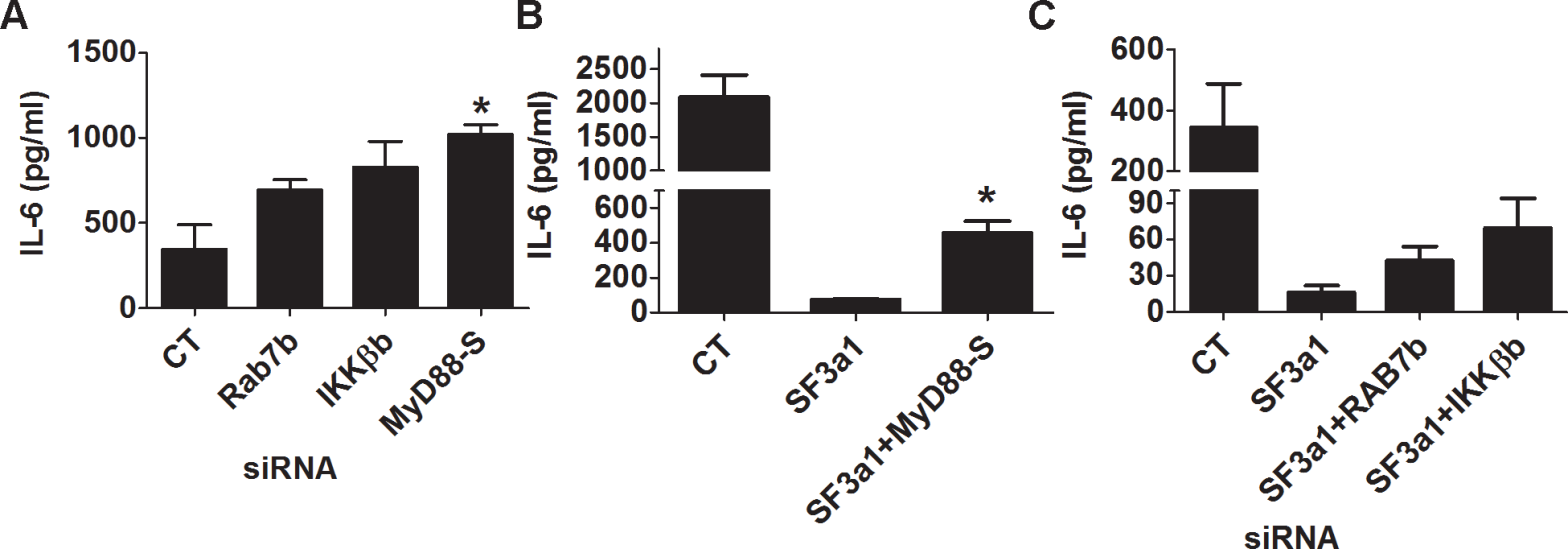
Multiple genes in the TLR signaling pathway mediate the effects of SF3a1 on innate immunity. RAW264.7 macrophages were treated with the indicated siRNAs or control non-targeting siRNA and subsequently exposed to 20 ng/ml LPS for 6 hours. IL-6 production was monitored by ELISA. In panels B and C, cells were treated with multiple siRNAs simultaneously, as indicated; in panel A, only a single siRNA was used in each case.

Our RNAseq analysis demonstrated that many TLR signaling pathway genes exhibit altered expression or mRNA splicing when SF3a1 was inhibited. This included a decrease in the production of several positively acting factors because of intron retention (CD14, IRAK1, and IKKβ) and an increase in production of several negatively acting factors from a variety of mRNA splicing changes (RAB7b, sTLR4, and possibly IKKβb). Additionally, using qPCR and RT-PCR, we previously demonstrated that SF3a1 inhibition led to an increase in production of the inhibitory splice form MyD88_S_ [26]. All of these changes in positively and negatively acting factors could contribute to the overall decrease in innate immune responsiveness caused by SF3a1 inhibition. To test the effect of several of these candidate negative regulators, we inhibited either of IKKβb, Rab7b, or MyD88_S_ using siRNA and found that all three treatments led to increased LPS-induced IL-6 production (Fig. 8A).

To verify that the effect of SF3a on innate immunity was mediated by these various factors, we used siRNA to simultaneously inhibit SF3a1 and these negatively acting isoforms. As described previously [46], inhibition of MyD88_S_ is able to partially rescue the effect of SF3a1 inhibition on LPS induced IL-6 production (Fig. 8B). Similarly, inhibition of Rab7b or IKKβb with siRNA each led to a small rescue of the effects of SF3a1 inhibition (Fig. 8C), suggesting that the effects of SF3a1 on innate immunity are mediated by altered splicing of multiple TLR signaling pathway genes.

### LPS stimulation and SF3a1 inhibition regulate the pre-mRNA splicing of a common gene set

**Figure 9:**
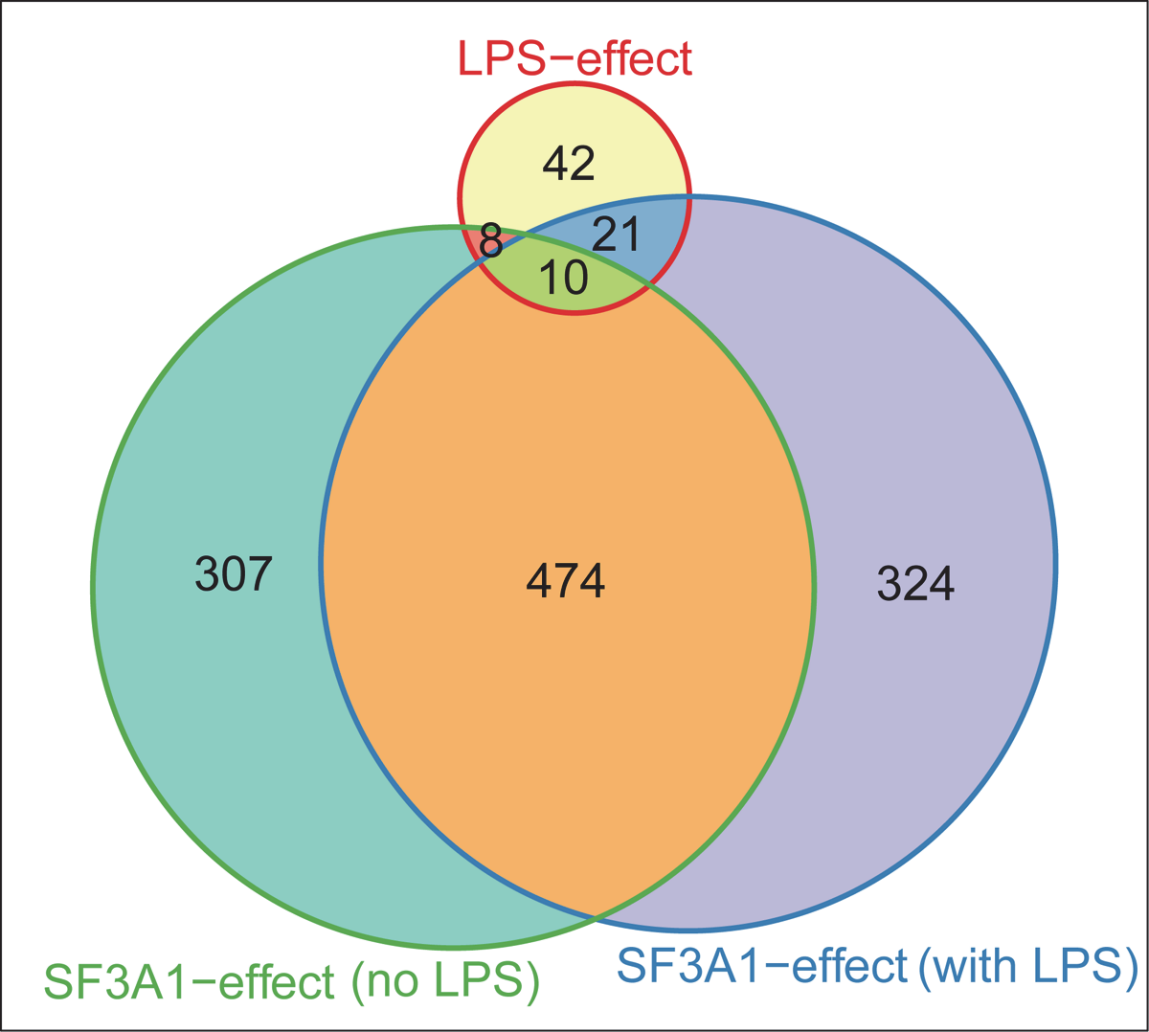
LPS challenge and SF3a1 inhibition affect pre-mRNA splicing of a common set of genes. The Venn diagram (generated using Vennerable package for R 3.0.1) indicates the number of genes whose splicing is altered in the DEXSeq analysis following LPS challenge alone, SF3a1 inhibition in the absence of LPS, or SF3a1 inhibition in the presence of LPS.

We found that LPS stimulation (Table 1) and SF3a1 inhibition (Table 2) both affected alternative pre-mRNA splicing of genes in innate immune signaling pathways. This suggested that specific alterations in the spliceosome may also influence specific effects of LPS on mRNA splicing in macrophages. To test this idea, we compared the lists of genes that were alternatively spliced in the DEXSeq analysis following LPS stimulation or SF3a1 inhibition and found, as expected from the MISO analysis (Fig. 2), that SF3a1 inhibition induced more alternative splicing events than did LPS stimulation (Fig. 9). More than half of all SF3a1-dependent alternative pre-mRNA splicing events were in the same 474 genes, regardless of LPS stimulation status (Fig. 9). A smaller set of differentially spliced genes (307), were observed with SF3a1 inhibition alone, and 324 differentially splice genes were unique to the combination of SF3a1 and LPS, consistent with a role for SF3a1 activity in modulating innate immunity regulation. Roughly half of the alternative gene splicing events specific to LPS stimulation alone (39/81) were also affected by SF3a1 inhibition (Fig. 9), suggesting that SF3a1 and the spliceosome exhibit some specificity in macrophages for regulating LPS-induced alternative splicing at this level of SF3a1 knockdown.

## Discussion

### Regulation of pre-mRNA splicing and disease

More than 95% of human genes are alternatively spliced [78–80], contributing to the complexity of the proteome. Cis-acting mutations that affect splicing of specific genes account for as much as 35% of inherited genetic disease [81–84]. Heritable mutations in splicing genes cause several rare diseases including spinal muscular atrophy, retinitis pigmentosa, Nager syndrome, mandibulofacial dysostosis, and oesophageal atresia [81–83,85–94]. Somatic mutations in splicing regulators also have been identified in various malignancies [95–106]. Thus, proper regulation of alternative splicing is critical for normal cellular functions and disease prevention.

While there have been reports of alternative pre-mRNA splicing in genes of the TLR signaling pathway, either globally [25] or on a gene by gene basis [15–20,23,24,26,107–112], there has been little study of how this alternative splicing is regulated. Our discovery that the TLR signaling pathway is particularly sensitive to perturbation of the core SF3a and SF3b spliceosome components in mouse and human macrophages [26] has provided an entry point for such a mechanistic study, and the current investigation of SF3a function has confirmed this surprising role of the core splicing machinery in regulation of the TLR signaling pathway in macrophages.

### The core SF3a mRNA splicing complex regulates alternative pre-mRNA splicing

As expected, SF3a1 inhibition affected a large number of splicing events, particularly intron retention and exon skipping. We note that these results may be biased as MISO examines only a subset of pre-identified possible alternative pre-mRNA splicing events [47]. Nevertheless, it is clear that when SF3a1 levels are reduced to 20% of their wild-type levels, the vast majority of mRNA splicing events still occur normally. It has been reported that the mRNA splicing machinery is limiting within the cell [113,114], so it is logical to assume that some genes will have splicing regulatory sequences that are more sensitive to spliceosomal perturbation than other genes. This partial specificity is not unexpected, as several studies demonstrate that inhibition or mutation of core splicing factors affects splicing of only a subset of genes [115–125], although the partial specificity of splicing factors for innate immune signaling pathways has not been noted previously. Moreover, the cis-acting regulatory sequences identified in this analysis are similar to those reported in other studies of the regulation of alternative pre-mRNA splicing [126,127]. Presumably in a complete knockout situation, many more mRNA splicing events would be affected. Consistent with this, inhibition of SF3a in HeLa cells affects cell survival [128]; this could reflect the stronger RNAi possible in HeLa cells or could be due to a cell-type specific effect. The possibility of cell-type specific effects of mRNA splicing factors are also raised by the report that SF3a1 functions with human estrogen receptor α to regulate mRNA splicing in other cell types [129].

### mRNA splicing and innate immunity

We found that both LPS stimulation and SF3a1 inhibition affected alternative splicing of a common set of genes in innate immune signaling pathways, suggesting that SF3a1 could play a role in mediating the effect of LPS on alternative pre-mRNA splicing. Inhibition of SF3a1 led to a decrease in production of several positive regulators of TLR signaling (intron retention in IRAK1, CD14, and IKKβ); SF3a1 inhibition also led to an increased production of negatively acting mRNA isoforms of TLR pathway genes (sTLR4, MyD88, Rab7b, and possibly IKKβ). Moreover, these negatively acting alternative isoforms are produced by a variety of alternative splicing events (sTLR4, exon inclusion; MyD88, exon skipping; IKKβ, intron retention; Rab7b, gene expression increase).

Why are these particular splicing events so sensitive to perturbation of the core spliceosome component SF3a1? An intriguing possibility is that perhaps, because of their functional significance, the mRNA splice site choices in these genes evolved to be key points of regulation to limit inflammation in macrophages. There is precedent for LPS or other components of pathogens altering the splicing machinery. For example, MyD88 activation in the presence of viral infection can decrease Polypyrimidine Tract Binding Protein (PTB) mRNA levels [130] (although PTB mRNA levels are not affected by LPS stimulation in our RNAseq data). LPS has been shown to stimulate phosphorylation of hnRNP A0, which binds to and stabilizes some cytokine mRNAs [131]. It is possible that LPS treatment could affect the activity of these or other components of the splicing machinery. It is possible that SF3a (or another component in the complex) could itself be modified by LPS stimulation. We have performed some preliminary tests to assess this possibility. SF3a1 subcellular localization (monitored using a SF3a1-GFP fusion) was not grossly altered following LPS stimulation. SF3a1 mRNA and protein levels did decrease slightly (to ∼70% of wild type levels) following LPS stimulation (monitored using qPCR and western blot), although it is unclear what the significance of this moderate decrease is. Conceivably, SF3a1 activity could also be modified by LPS treatment through some covalent modification.

Future investigations of these specific splicing events will inform us how this alternate splicing is regulated and if these splicing events are regulated by a single common mechanism or if multiple independent mechanisms regulate alternative splicing in the TLR signaling pathway. Harnessing these regulatory mechanisms to alter mRNA splicing in the TLR signaling pathway could prove to be a useful novel approach to modulate inflammation, thereby treating numerous inflammatory diseases.

## Materials and Methods

### RNAseq analysis to monitor the effect of SF3a1 inhibition

The RAW264.7 mouse macrophage cell line was transfected with either SF3a1 siRNA or control non-targeting siRNA (Dharmacon) using the 96-well shuttle transfection system (Amaxa) as described previously [26]. Twenty-four hours later, cells were exposed to either 20 ng/ml LPS (List Biological labs), or not as a control. All RNAi treatments and exposures were performed in triplicate. Four hours after LPS stimulation, the supernatant was removed for ELISA analysis and the cell pellet was lysed in RLT buffer (Qiagen). Total RNA was purified using the RNAeasy kit (Qiagen). A portion of the RNA was set aside for qPCR analysis, and the remainder was purified further for RNAseq. PolyA-RNA was isolated from total RNA using the Dynabeads mRNA Direct Purification kit (Life Technologies). The polyA RNA was then processed for next-generation sequencing (NGS) library construction following standard procedures for Ion Proton sequencing using the Ion Total RNA-seq kit for whole transcriptome libraries (Life Technologies). Briefly, library construction proceeded from adaptor ligation, to reverse transcription, cDNA size selection and amplification, and finally bead templating. Once validated, the libraries were sequenced as barcoded-pooled samples on multiple Ion P1 chips using an Ion Proton NGS platform. The RNAseq data presented in this article have been deposited in the Gene Expression Omnibus database (http://www.ncbi.nlm.nih.gov/geo/) under accession number GSE58432.

### Mapping of sequences to the genome

The gene model used throughout these analyses is based on the Ensembl annotation downloaded July 29, 2013, from the UCSC Genome Browser (http://genome.ucsc.edu/). Sequence reads 30 nucleotides long or greater were mapped to the UCSC release mm10 of the *Mus musculus* genome using GSNAP version 2013-11-27 (http://research-pub.gene.com/gmap/ [132,133]) with SNP data version 137 and splice sites compatible with the Ensembl annotation, as well as detection of novel splice sites. With the exception of the Cufflinks/Cuffdiff analysis, only uniquely mapping reads were used for further analysis; multiply-mapped reads without translocations were added for the Cufflinks/Cuffdiff analysis. A separate set of alignments was generated for analysis with MISO, which requires a fixed read length. For MISO, sequences were truncated to 70 nt (replicate 1) or 60 nt (replicates 2 and 3) and shorter reads discarded prior to mapping with GSNAP.

### Analysis of differential splicing with MISO

Genes with splicing events that differ significantly between treatments were identified with MISO version 0.4.9 (http://genes.mit.edu/burgelab/miso/ [47]), using version 2 of MISO’s annotation of alternative splicing events for UCSC release mm10. Because MISO cannot take into account replicates, we treated an event as significantly different between treatments if the change was in the same direction for all three replicates and the Bayes factor of each was at least 1. Events were considered unchanged between treatments if all three Bayes factors were less than 1.

### Analysis of cis-acting splicing regulatory sequences

To identify potential differences in the splice sites of genes with and without changes in splicing, we created sequence logos [134] of those sites with WebLogo version 3.3 (http://weblogo.threeplusone.com/ [135]). Based on the logos of the 3′-splice site, we compared the base composition of the polypyrimidine tract region extending from positions −17 to −5 counting from the 3’ splice site. Fractions of each base in each intron of one set (e.g. introns significantly more retained in SF3a1-depleted cells than in control cells) were compared to a control set (e.g. introns that showed no change, regardless of SF3a1 levels) using the nonparametric Mann–Whitney U-test (wilcox.test function in the stats package of R version 3.0.1 [136]). For intron-retention events, we compared introns that were significantly more retained when SF3a1 was inhibited to introns whose retention was not altered by SF3a1 inhibition (as identified by MISO, see above). For exon skipping events, we compared 3′-splice site sequence logos for the introns both upstream and downstream of the potentially skipped exon.

To determine if exon and intron lengths differed significantly between the various conditions, the nonparametric Mann–Whitney U-test (wilcox.test function in the stats package of R version 3.0.1) was used.

### Differential gene expression analysis with DESeq

Reads mapping to each gene in the Ensembl annotation were quantified using the htseq-count program from the HTSeq package version 0.5.4p3 in the “intersection-nonempty” mode (http://www-huber.embl.de/users/anders/HTSeq/ [137]). These counts were analyzed for differential expression with the DESeq package version 1.12.1 [48] under R version 3.0.1, using a false discovery rate (FDR) of 0.1.

### Differential exon expression analysis with DEXSeq

To examine changes in splicing based on differential exon expression, we used the DEXSeq package version 1.6.0 [49] under R version 3.0.1 with an FDR of 0.1. Exon counts for this analysis were obtained with the included HTSeq-based script dexseq_count.py and an annotation based on the Ensembl gene model.

### Differential gene and transcript expression analysis with Cufflinks

Cufflinks version 2.1.1 [50,51,138,139] was used to assemble and quantify transcripts with parameters to mask rRNA and tRNA sequences and enable bias correction and multi-mapped read correction, and without a reference annotation. Other cufflinks parameters were as follows: -j 0.1 -A 0.05 —overhang-tolerance 5 —max-bundle-length 5,000,000. Transcript models from the different samples and replicates were combined using cuffmerge with the Ensembl annotation and the mouse mm10 genome sequence as references. Testing for differences in gene expression and splicing was performed using cuffdiff with bias correction and multi-mapped read correction, as well as masking of rRNAs and tRNAs, using the default FDR of 0.05. Since three replicates were available for each treatment, dispersion was estimated separately for each condition.

### Pathway analysis

To determine which pathways were altered by LPS treatment or SF3a1 inhibition, the genes identified in the DEXseq analysis were analyzed using the GATHER utility in network mode [52] or the DAVID utility [53,54].

### qPCR to monitor isoform-specific mRNA levels

qPCR was performed using the Quantitect SYBR-green RT-PCR assay kit (Qiagen) and an ABI 7900 thermocycler. Data was normalized relative to β-actin, whose splicing is not affected by this level of SF3a1 inhibition [26]. Primer sequences used for qPCR are listed in S21 Table. qPCR was performed in triplicate and analyzed with Graphpad Prism 5 using t-tests to determine statistical differences (p<0.05).

### Gene inhibition using siRNA

RAW264.7 or J774A.1 mouse macrophages were tranfected with siRNAs (Dharmacon, either SMARTpools targeting particular genes or non-targeting control pools) using the Amaxa nucleofector Shuttle (Lonza) as described previously [26,27,46]. Cells were then plated in 96-well format (100,000 cells per well). Twenty-four hours later, cells were stimulated with 20 ng/ml LPS for six hours, supernatant was collected for ELISA (R&D Biosystems) and cell pellets were used to monitor viability with fluorescein diacetate [140,141] and lysed in RLT (Qiagen) buffer to prepare RNA for qPCR (which was performed as described above). In experiments using two siRNAs simultaneously, siRNA treatments containing only one siRNA were supplemented with a second negative control non-targeting siRNA to render the volumes equivalent.

Sequences of the siRNAs targeting IKKβ intron 15 were 5′-AAGCAGAAGUCUCAGGAUA(UU)-3′ and 5′-GGGCAGAGUUGCUCCGGAU(UU)-3′. ELISA experiments were performed in triplicate and analyzed using Graphpad Prism 5 using t-tests to determine statistical differences (p<0.05).

### Western blot to monitor protein levels

RAW264.7 cells were transfected with either SF3a1-specific siRNA or control non-targeting siRNA as described above. Following the siRNA treatment, cells were lysed on ice in RIPA buffer supplemented with protease inhibitors. Lysates were centrifuged at 12,000 RPM for 15 minutes at 4°C, protein concentration of the supernatant was assessed by BCA Assay (Pierce), and samples were boiled in SDS-loading buffer. Samples were separated on 10% SDS-polyacrylamide gels and transferred to nitrocellulose. The membranes were blocked for 2 hours at room temperature in TBS-T containing 5% non-fat milk, incubated overnight at 4°C with primary antibodies (1:1000) in TBS-T plus 5% BSA (rabbit-anti-IRAK1 and rabbit-anti-IKKβ antisera were from Cell Signaling Technology; mouse-anti-β-actin antiserum was from Millipore), washed in TBS-T, then incubated with HRP-conjugated secondary antibodies (1:1000) for 1 hour at room temperature. The membrane was then washed, treated with ECL Substrate (Pierce), and fluorescence was captured by autoradiography. Images of the films were captured with a Nikon D200 camera. Bands were quantified using Image J [142] and subsequently analyzed for significant differences in Graphpad Prism 5 using t tests (p<0.05).

## Supporting Information

S1 Fig SF3a1 inhibition decreases production of IRAK1 and IKKβ protein. RAW264.7 cells were subjected to either SF3a1 siRNA or control siRNA (CT), were exposed to 20 ng/ml LPS for six hours (IKKβ) or were not exposed to LPS (IRAK1), and IRAK1 and IKKβ protein levels were monitored by western blot. Panels A and B display western blots depicting representative production of IRAK1 and IKKβ (top) and the same blots re-probed for β-actin (bottom). Panels C and D depict quantitation of IRAK1 and IKKβ levels, respectively, from three independent experiments. Asterisks indicate results that were statistically different than control (t-test, p<0.05).
(PDF)

S2 Fig SF3a1 inhibition alters mRNA splicing of TLR signaling pathway genes in the mouse J774A.1 cell line. Panels A–H: J774A.1 cells were subjected to either SF3a1 siRNA or control siRNA (CT), were exposed to 20 ng/ml LPS for six hours, and qPCR was used to to monitor the production of the indicated mRNA isoforms (expression normalized so that 1 is the expression in the presence of control siRNA). CT indicates control siRNA, SF3a1 indicates SF3a1-specific siRNA. LPS exposures were performed for six hours in the presence of 20 ng/ml LPS. Asterisks indicate results that were statistically different than control (t-test, p<0.05).
(PDF)

S1 Table Retained Introns (MISO) – CT siRNA with LPS vs SF3a1 siRNA with LPS.
(XLSX)

S2 Table Skipped Exons (MISO) – CT siRNA with LPS vs SF3a1 siRNA with LPS.
(XLSX)

S3 Table Alternate 3’ Splice Site (MISO)—CT siRNA with LPS vs SF3a1 siRNA with LPS.
(XLSX)

S4 Table Alternate 5’ Splice Site (MISO)—CT siRNA with LPS vs SF3a1 siRNA with LPS.
(XLSX)

S5 Table Mutually Exclusive Exons (MISO)—CT siRNA with LPS vs SF3a1 siRNA with LPS.
(XLSX)

S6 Table DESeq – CT siRNA no LPS vs CT siRNA with LPS.
(XLSX)

S7 Table DESeq – CT siRNA no LPS vs SF3a1 siRNA no LPS.
(XLSX)

S8 Table DESeq – CT siRNA with LPS vs SF3a1 siRNA with LPS.
(XLSX)

S9 Table DEXSeq – CT siRNA no LPS vs CT siRNA with LPS.
(XLSX)

S10 Table DEXSeq – CT siRNA no LPS vs SF3a1 siRNA no LPS.
(XLSX)

S11 Table DEXSeq – CT siRNA with LPS vs SF3a1 siRNA with LPS.
(XLSX)

S12 Table Cuffdiff GENE EXP—CT siRNA with LPS vs SF3a1 siRNA with LPS.
(XLSX)

S13 Table Cuffdiff CDS EXP—CT siRNA with LPS vs SF3a1 siRNA with LPS.
(XLSX)

S14 Table Cuffdiff Isoform EXP—CT siRNA with LPS vs SF3a1 siRNA with LPS.
(XLSX)

S15 Table Cuffdiff TSS group EXP—CT siRNA with LPS vs SF3a1 siRNA with LPS.
(XLSX)

S16 Table Cuffdiff CDS—CT siRNA with LPS vs SF3a1 siRNA with LPS.
(XLSX)

S17 Table Cuffdiff Promoters—CT siRNA with LPS vs SF3a1 siRNA with LPS.
(XLSX)

S18 Table Cuffidff Splicing—CT siRNA with LPS vs SF3a1 siRNA with LPS.
(XLSX)

S19 Table MISO Global Splicing Analysis.
(XLSX)

S20 Table Polypyrimidine tract analysis.
(XLSX)

S21 Table Oligonucleotide sequences.
(XLSX)
