# Supplementary figures and images for: Regulation of Toll-like Receptor Signaling by the SF3a mRNA Splicing Complex
Source: PLoS Genet. 2015 Feb 6;11(2):e1004932. doi: 10.1371/journal.pgen.1004932 (PMC4450051; doi:10.1371/journal.pgen.1004932)

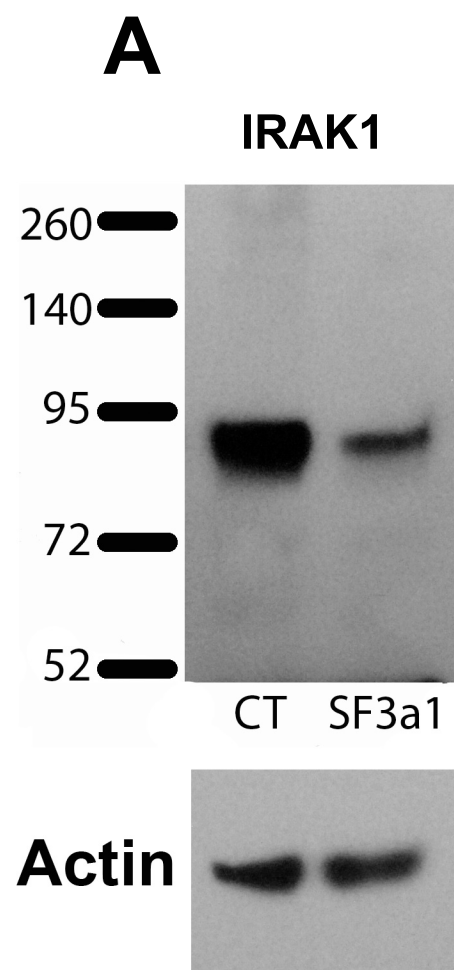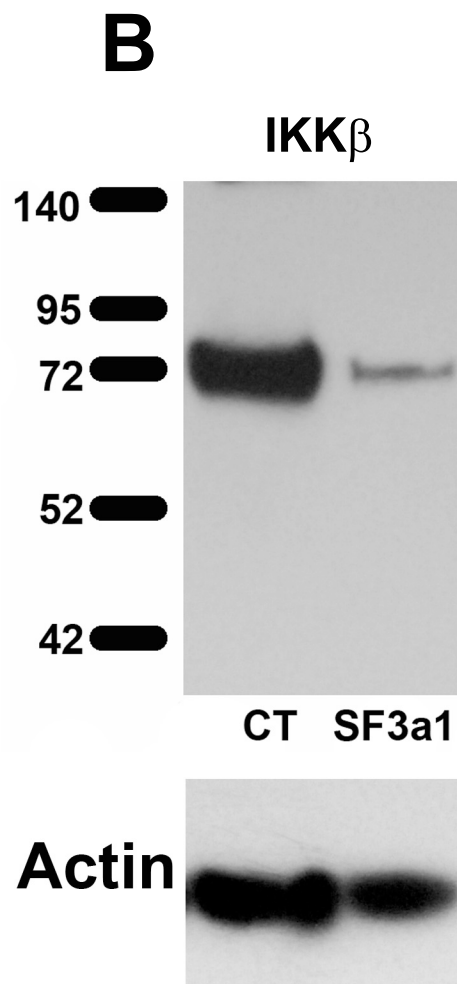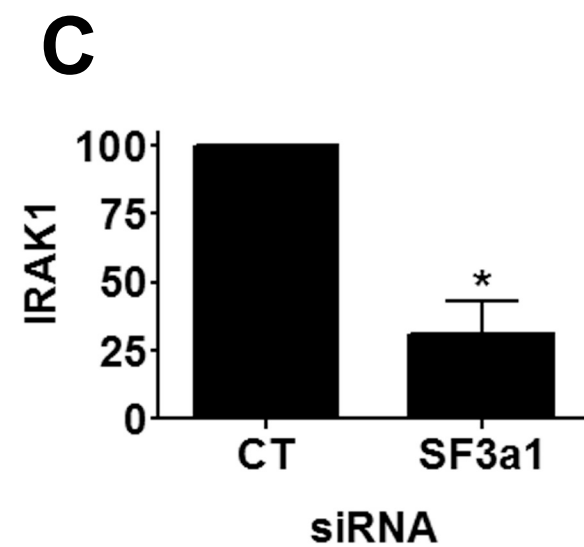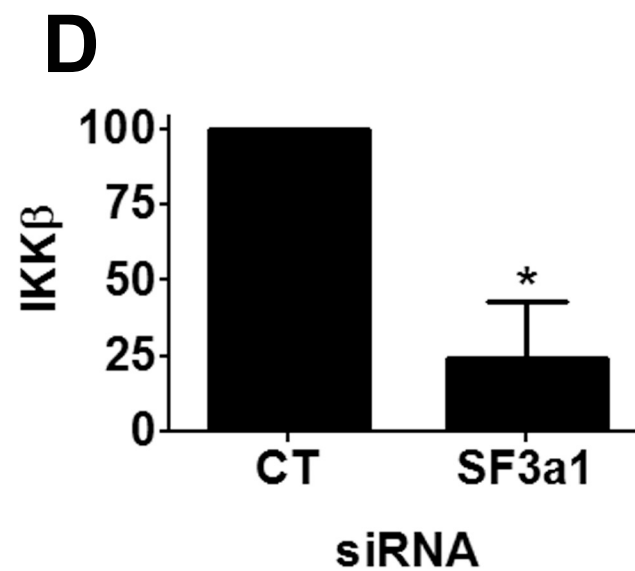

Supplement: S1 Fig — RAW264.7 cells were subjected to either SF3a1 siRNA or control siRNA (CT), were exposed to 20 ng/ml LPS for six hours (IKKβ) or were not exposed to LPS (IRAK1), and IRAK1 and IKKβ protein levels were monitored by western blot. Panels A and B display western blots depicting representative production of IRAK1 and IKKβ (top) and the same blots re-probed for β-actin (bottom). Panels C and D depict quantitation of IRAK1 and IKKβ levels, respectively, from three independent experiments. Asterisks indicate results that were statistically different than control (t-test, p<0.05). (PDF) [file pgen.1004932.s001.pdf]

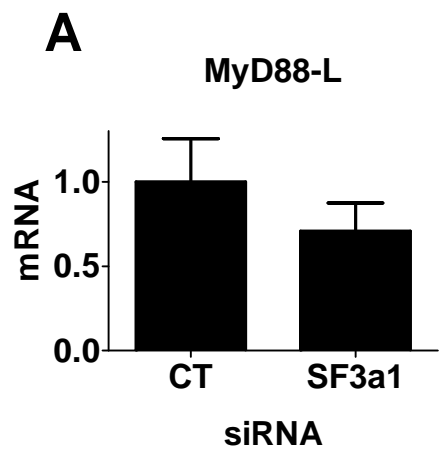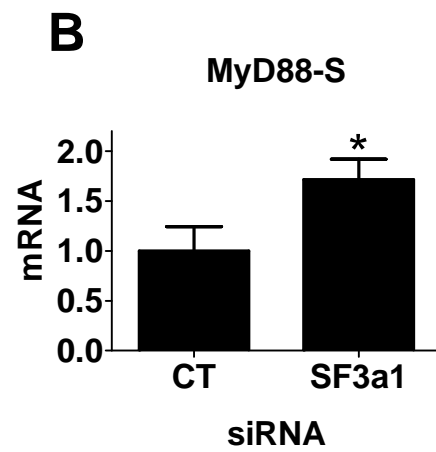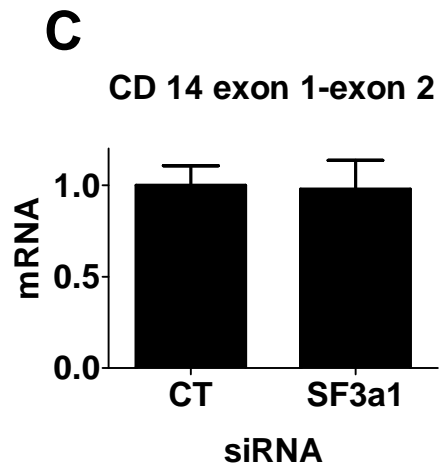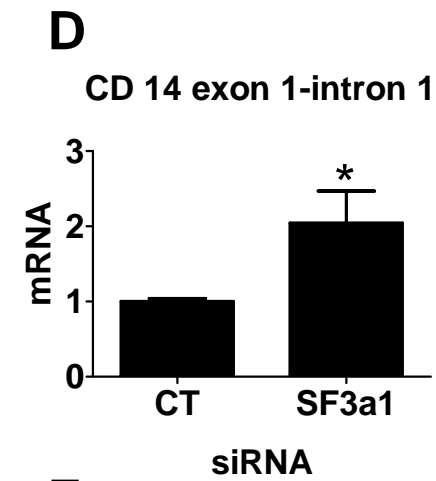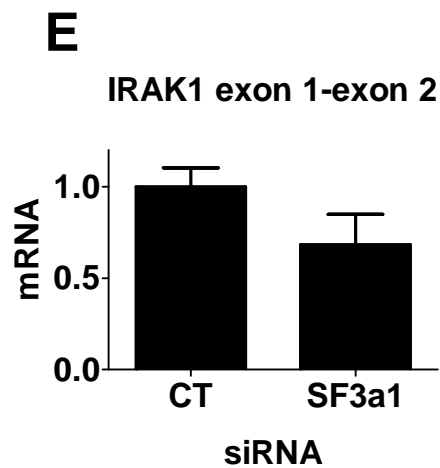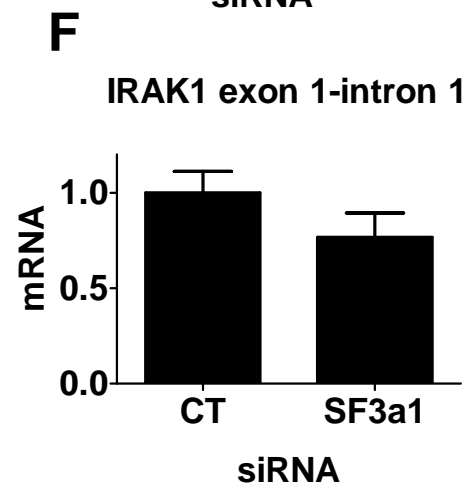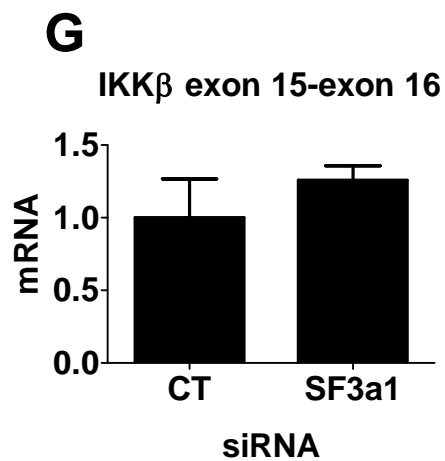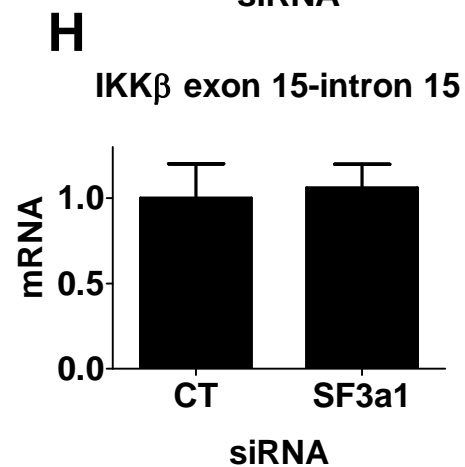

Supplement: S2 Fig — Panels A–H: J774A.1 cells were subjected to either SF3a1 siRNA or control siRNA (CT), were exposed to 20 ng/ml LPS for six hours, and qPCR was used to to monitor the production of the indicated mRNA isoforms (expression normalized so that 1 is the expression in the presence of control siRNA). CT indicates control siRNA, SF3a1 indicates SF3a1-specific siRNA. LPS exposures were performed for six hours in the presence of 20 ng/ml LPS. Asterisks indicate results that were statistically different than control (t-test, p<0.05). (PDF) [file pgen.1004932.s002.pdf]
